# Fungal aerosol and particulate matter in horse stables in Poland

**DOI:** 10.1007/s00253-024-13258-4

**Published:** 2024-07-24

**Authors:** Jacek Grzyb, Zenon Podstawski, Karol Bulski

**Affiliations:** 1 Department of Microbiology and Biomonitoring, University of Agriculture in Krakow, Mickiewicza Ave 24/28, 30-059 Kraków, Poland; 2 Department of Reproduction, Anatomy and Genomics of Animals, University of Agriculture in Krakow, Mickiewicza Ave 24/28, 30-059 Kraków, Poland

**Keywords:** Horse stable, Bioaerosol, Fungal aerosol, Particulate matter, Size distribution

## Abstract

**Abstract:**

Horses stay in different types of stables; especially during the cold season, they stay inside for most of the day. A stable is also a place where many people spend quite a lot of time either as employees who care for and train horses or as equine enthusiasts. Keeping horses in stables causes their constant exposure to high concentrations of particulate matter (PM) and molds in the air inside these facilities. The study was conducted in Udórz Stud Farm located in the southern region of Poland. It was carried out in two different types of stables: three runners and two box stables. The study continued for 2 years; samples were collected in each season of the year. The following devices were used: a six-stage Andersen-Graseby cascade impactor, the DustTrak™ II Aerosol Monitor 8530. The obtained results allowed for the conclusion that horses kept in box stables are exposed to lower concentrations of molds and yeasts than those kept in runners. Molds dominated in the stable air during humid periods—spring and autumn—while yeasts were more prominent during summer and winter. It was observed that cleaning stables reduces the morphotic elements of fungi in the air, even though it results in a higher level of particulate matter in the stable air. It should be noted that microclimate conditions were optimal for horses practically throughout the whole year.

**Key points:**

*• In stables, there is a high level of air intoxication, both by yeast and by mold fungi*

*• The concentrations of fungi in the air depend on the season and the stable cleaning procedure*

*• The PM concentrations depend on the type of stable*

## Introduction

In recent years, a significant increase in the incidence and severity of equine respiratory system disorders has been observed. This is the result of keeping horses in stables and riding them in closed rooms, which causes them to be constantly exposed to high concentrations of particulate matter (PM) in the air inside these facilities. Although horses are most often taken outside the stable for cleaning and feeding, the sedimentation rate of fine particles is so low that PM concentration is still high after horses return to the stable. PM in stables comes from bedding, feed, dried faeces, mites, and exfoliated animal cells; hence, up to 70% of PM is of organic origin (Mostafa et al. 2021). Particulate matter is a carrier for biological particles; hence, its concentration is correlated with the number of microorganisms creating bioaerosols (Nazarenko et al. 2018; Wolny-Koładka 2018; Lenart-Boroń et al. 2022). The level of particulate matter in the stable air is affected by many factors, such as the type of building, type and efficiency of ventilation, facility size, livestock density, type of feed, type of bedding, microclimatic conditions inside the stable, and climate where the stables are located (Witkowska et al. 2012; Siegers et al. 2018; Perrin 2021). Plant products used in stables—hay as feed and straw as bedding—contain a significant amount of autochthonous microflora, which can multiply in unfavourable storage conditions, so moldy feed and low-quality bedding become significant sources of mold spores. The size of the spores allows them to penetrate up to the level of the lung alveoli, where they may have infectious, toxic, and allergenic effects separately or in combination (Woolnough et al. 2015; Rick et al. 2016).

Due to their size, both dust particles and bioaerosol components can be divided into two fractions: total, with an aerodynamic diameter above 4.7 µm, which can settle in the upper respiratory tract, and respirable, which includes particles with a diameter below 4.7 µm. Particles with a diameter of 3.3–4.7 μm reach the trachea and primary bronchi, particles 1.1–3.3 μm reach the secondary bronchi and bronchioles, while particles smaller than 1.1 μm may reach the alveoli (Górny and Dutkiewicz 2002). The exact size of particles penetrating particular levels of the respiratory tract in horses is unknown. However, it is assumed that the abovementioned particles belonging to the respirable fraction reach the same place in the respiratory tree of humans, horses, and other animals (Hessel et al. 2009; Auger and Moore-Colyer 2017; Claußen and Hessel 2017).

This study aimed to determine how the concentrations of mold fungi and yeasts are influenced by different horse management systems, changing seasons and PM concentrations inside the stable. The obtained study results will be used to determine potential health threats to horses, grooms and equine enthusiasts.

## Materials and methods

The study was carried out in Udórz Stud Farm. The facility is located in southern Poland. Four stables are located within the stud farm area. The first building includes two runner stables (R1 and R2), the second one another runner (R3), and two adjacent buildings include box stables (B1 and B2). The reference testing station (control) was located on the stud farm’s premises, approximately 50 m from the nearest stable. While the study was conducted, there were 84 horses in all stables. The characteristics of the stables and horses are presented in the article by Grzyb et al. (2022).

The measurements were taken over two calendar year periods—one measurement per each season of the year. The following months were selected as the most representative for a given season: April (spring), July (summer), September (autumn), and February (winter).

Bioaerosol samples were taken using a six-stage cascade impactor WES-710 model Andersen-Graseby (Westech Scientific Instrument, Hatfield Peverel, Essex, Great Britain). This impactor enables to divide bioaerosol stream into fractions based on the aerodynamic diameters of the particles: above 7 µm (F1); 4.7–7 µm (F2); 3.3–4.7 µm (F3); 2.1–3.3 µm (F4); 1.1–2.1 µm (F5), and 0.65–1.1 µm (F6). Fractions below 4.7 µm (F3-F6) are classified as respirable (RF).

The first measurement series were taken between 7:00 a.m. and 10:00 a.m.—before feeding and cleaning the stable (“Before bedding” (BB)), and the second series, after cleaning the stables, feeding horses, and adding fresh bedding (11:00 a.m.–2:00 p.m.) (“After bedding” (AB)).

Taking into account the location of the breathing zone of people and horses, air samples were collected 1.5 m above the ground (floor). The airflow rate through the impactor was constant and amounted to 28.3 dm^3^ per minute. The time for collecting air samples inside the stable ranged from 10 to 40 s; i.e., the volume of air passing through the impactor ranged from 4.7 to 18.9 dm^3^; at the control station (outside the stable), it took from 120 to 240 s (56.6–113.2 dm^3^).

MEA growth medium (Malt Extract Agar, Biomaxima, Lublin, Poland) was used for culturing fungi. The media were incubated for 5–7 days at 28 °C under aerobic conditions. After the incubation, colonies in Petri dishes were counted. Mold and yeast concentrations were calculated according to the following formula: *L* = [Pr·1000]/*v*, where *L* is the concentration of microorganisms in 1 m^3^ of air, Pr is the probable count of colonies according to the impactor manufacturer’s table, *v* is the volume of air taken by the impactor (dm^3^), 1000 is the converter to 1 m^3^. The results were presented as colony-forming units per 1 m^3^ of air (CFU/m^3^). The tests were taken in triplicate and the results were presented as medians of concentrations.

There are no guidelines regarding permissible concentrations of microorganisms in farm facilities, including stables. The obtained results were compared to the proposals of the Team of Experts in Biological Factors (ZECB) (Augustyńska and Pośniak 2016) on the recommended concentrations of microorganisms, treating stables as work premises contaminated with biological particulate matter (Table 1).

**Table 1:** Proposals for acceptable concentrations of fungi according to the Team of Experts in Biological Factors (ZECB)

Microbiological agent	Acceptable concentration [CFU/m^3^]
Total count of fungi (TC)	50,000
Respirable fraction of fungi (RF)	25,000

The DustTrak™ II Aerosol Monitor 8530 (TSI Inc., Shoreview, MN, USA) has interchangeable heads enabling the measurement of four fractions of particulate matter: PM_10_, PM_4_, PM_2.5_, and PM_1_ (i.e., PM particles with a diameter below 10, 4, 2.5, and 1 μm, respectively). The sampling time for each PM fraction was 180 s, and a single concentration reading was taken every 3 s, which gave a total of 60 independent measurements of average particulate matter concentrations.

Statistical analysis of the data was performed using the computer program Statistica data analysis software system, version 13.0 (TIBCO Software Inc., Santa Clara, CA, USA). After taking into account the fulfilment of the assumptions about the normality of the distribution of variables (Shapiro–Wilk’s test) and the homogeneity of variance (Levene’s test), the significance of differences between the means was verified with Kruskal–Wallis test. The values for which the probability of “*p*” was lower than 0.05 were considered statistically significant. The impact of particulate matter on the quantitative presence of fungi (molds and yeast) in the air was assessed using Spearman’s rank correlation coefficient, assuming statistically significant values at *p* < 0.05.

## Results

The obtained results were compared to the ZECB recommendations, and stables were treated as work premises contaminated with biological particulate matter. As presented in Table 2, 30% of air samples from the tested stables exceeded the recommended total fungal aerosol concentration (TC), while in the case of the respirable fraction (RF), as many as 55% of samples. The type of stable (runner vs. box) did not have a significant impact on the number of times the recommended concentrations were exceeded. Significantly more measurements with recommended concentrations exceeded were taken BB as compared to AB. Interestingly, a significantly higher number of exceedances was recorded before cleaning the stables: 55% for TC and as much as 85% for RF. After cleaning the stables, these indicators were 11 times lower for TC and 3.4 times for RF, respectively, compared to the measurements taken before cleaning.

**Table 2:** Share of measurements exceeding the permissible concentration [in %] of molds and yeasts (TC) in the stable air in relation to the ZECB guidelines

Factor		Fraction	
		TC	RF
Total		30	55
Stable	All R	31	54
	All B	28	56
Bedding	AB	55	85
	BB	5	25
Season	Spring (April)	30	65
	Summer (July)	40	60
	Autumn (September)	15	45
	Winter (February)	35	50

*BB* before bedding, *AB* after bedding, *all R* runners, *all B* box stable, *C* control

It was observed that the median concentration of mold fungi in the stable air, regardless of the type of fungi, was more than twice as high before the stable cleaning as compared to the stable after cleaning (Table 3). In turn, the median fungal aerosol concentration for two types of stables, i.e., running stable (all R) and box stable (all B), before cleaning was almost identical and amounted to approximately 23,000 CFU/m^3^. When the stables were cleaned, a slightly higher median was observed in the case of box stables.

**Table 3:** Median, standard deviation, range, and total molds aerosol concentrations in stables [CFU/m^3^]—before and after bedding

Stable	Bedding	Median ± standard deviation	Range	Total
All R	BB	23,405 ± 51,395	1166–255,777	887,530
	AB	10,867 ± 32,052	797–162,392	437,364
All B	BB	23,410 ± 22,182	3969–81,432	467,099
	AB	11,713 ± 13,278	1699–43,198	247,499
C		1546 ± 3141	703–10,176	20,583

*BB* before bedding, *AB* after bedding, *all R* runners, *all B* box stable, *C* control

Table 4 presents data on yeast concentrations in the stable air. Based on median concentrations, it was found that, unlike molds, higher yeast concentrations occurred in box stables (all B), both before and after cleaning. Compared to the runners, the concentration differences were several per cent higher. As in the case of mold fungi, higher concentrations of yeast in the air occurred in stables BB. Compared to mold, the difference in the concentration of yeast in the air inside the stable buildings in comparison to the control station was even higher and ranged from 8 to 58 times.

**Table 4:** Median, standard deviation, range, and total yeasts aerosol concentrations in stables [CFU/m^3^]—before and after bedding

Stable	Bedding	Median ± standard deviation	Range	Total
All R	BB	18,353 ± 123,851	530–392,304	1,855,200
	AB	4690 ± 18,357	1378–90,028	270,244
All B	BB	21,244 ± 19,855	5090–75,721	399,665
	AB	5486 ± 4949	1768–15,549	111,164
C		452 ± 808	220–2663	6926

*BB* before bedding, *AB* after bedding, *R* runners, *B* box stable, *C* control

Using the Andersen-Graseby cascade impactor, the particle size distribution of fungal aerosol was also examined (Tables 5 and 6) in the tested farm facilities. It was found that the highest median mold concentrations were in the fine fraction F5 (2.2–1.1 µm) in both types of stables—before as well as after cleaning (Table 5). The lowest concentrations in the box stables (both BB and AB) and runners (AB) were of the ultrafine fraction penetrating the deepest into the respiratory system of both humans and horses (F6). In the runners before cleaning (BB), the thickest fraction F1 had the smallest share. Each of the fungal aerosol fractions in the air showed higher concentrations during measurements taken before cleaning the stables (BB); the difference ranged from 23 to 75% compared to AB measurements. The minimum and maximum mold concentrations were recorded at the control station (C) for fractions other than those inside the stable: fraction F4 had the highest concentrations and F2 the lowest. This was because different types of fungi, with different spore sizes, dominate in atmospheric air, and different ones in the air inside the stables. Yeast showed the highest concentrations and the largest share in the case of fraction F4 (Table 6): in the runners (before and after cleaning), in the box stable after cleaning and at the control station. Only in the case of the box stable before cleaning, the highest share of fraction F5 was recorded. The lowest proportion of yeast was found in fraction F6 in both types of stables, both AB and BB. At the control station, fraction F2 had the smallest share. It was observed that, as in the case of mold, higher yeast concentrations were also recorded during measurements taken before cleaning the stables; the differences ranged from 57 to 80% depending on a fraction.

**Table 5:** Median, standard deviation, and range of aerosol of molds in stables [CFU/m^3^]—split by fraction

	Fraction [µm]	Median ± standard deviation	Range (min–max)	Median ± standard deviation	Range (min–max)	Change of concentrations—BB vs AB* [%]
All R		BB		AB		
	F1	1844 ± 4560	0–21,563	1204 ± 1647	71–7102	− 34%
	F2	2872 ± 7959	141–39,650	734 ± 3208	71–15,688	− 74%
	F3	4440 ± 7152	424–36,520	1219 ± 5211	53–26,288	− 73%
	F4	3857 ± 19,936	318–99,874	2373 ± 17,519	230–87,768	− 38%
	F5	4804 ± 12,420	212–54,590	3712 ± 5061	106–19,398	− 23%
	F6	2497 ± 3851	0–19,630	618 ± 2399	18–10,888	− 75%
	TC	23,405 ± 51,395	1166–255,777	10,867 ± 32 052	797–162,392	− 54%
	RF	18,533 ± 39,681	954–194,564	7705 ± 27,736	655–139,602	− 58%
All B		BB		AB		
	F1	2482 ± 1823	558–5582	1069 ± 904	212–3110	− 57%
	F2	3155 ± 3095	750–11,540	1130 ± 1595	142–6292	− 64%
	F3	4210 ± 2663	655–8904	1484 ± 1848	71–5585	− 65%
	F4	4622 ± 5111	559–18,500	2686 ± 2891	71–9050	− 42%
	F5	5484 ± 9229	496–28,630	3454 ± 6084	212–19,584	− 37%
	F6	1696 ± 3175	94–10,450	967 ± 1702	71–7141	− 43%
	TC	23,410 ± 22,182	3969–81,432	11,713 ± 13,278	1699–43,198	− 50%
	RF	19,242 ± 17,835	2661–66,336	10,217 ± 11,298	919–33,936	− 47%
Control (C)	F1	182 ± 766	71–2332			
	F2	124 ± 192	47–636			
	F3	257 ± 226	141–848			
	F4	395 ± 806	57–2544			
	F5	270 ± 585	78–1696			
	F6	267 ± 677	62–2120			
	TC	1547 ± 3141	703–10,176			
	RF	1238 ± 2222	370–7208			

^*^Computed in relation to the median; fractions: F1 = 11.0–7.0 µm, F2 = 7.0–4.7 µm, F3 = 4.7–3.3 µm, F4 = 3.3–2.1 µm, F5 = 2.2–1.1 µm, F6 = 1.1–0.65 µm

**Table 6:** Median, standard deviation, and range of aerosol of yeasts in stables [CFU/m^3^]—split by fraction

	Fraction [µm]	Median ± standard deviation	Range (min–max)	Median ± standard deviation	Range (min–max)	Change of concentrations—BB vs AB* [%]
All R		BB		AB		
	F1	1947 ± 19,750	0–64,959	779 ± 1186	71–5406	− 60%
	F2	3310 ± 19,057	106–75,200	460 ± 751	71–2438	− 86%
	F3	2883 ± 24,092	0–89,622	1071 ± 9098	212–45,582	− 63%
	F4	3536 ± 29,389	0–93,210	1141 ± 7868	283–39,263	− 68%
	F5	3091 ± 27,839	106–122,536	1070 ± 2604	106–10,282	− 65%
	F6	1367 ± 16,809	0–52,640	328 ± 525	0–1696	− 76%
	TC	18,353 ± 123,851	530–392,304	4690 ± 18,357	1378–90,028	− 74%
	RF	13,352 ± 89,635	424–261,884	3640 ± 17,674	1060–87,756	− 73%
All B		BB		AB		
	F1	1810 ± 1534	362–4850	778 ± 540	141–2120	− 57%
	F2	1657 ± 2276	283–8547	638 ± 803	0–3100	− 61%
	F3	3044 ± 3681	0–14,520	687 ± 1275	212–5183	− 77%
	F4	4581 ± 11,481	707–46,516	1169 ± 1762	354–6532	− 74%
	F5	4795 ± 5807	1112–21,045	954 ± 1712	71–6148	− 80%
	F6	992 ± 2725	283–10,812	247 ± 422	0–1186	− 75%
	TC	21,244 ± 19,855	5090–75,721	5486 ± 4949	1768–15,549	− 74%
	RF	17,148 ± 17,763	3642–71,313	4015 ± 3978	778–12,425	− 77%
Control (C)	F1	77 ± 173	0–518			
	F2	57 ± 90	26–298			
	F3	116 ± 121	24–341			
	F4	124 ± 283	35–910			
	F5	66 ± 99	0–282			
	F6	76 ± 125	9–376			
	TC	452 ± 808	220–2663			
	RF	342 ± 576	141–1846			

^*^Computed in relation to the median; fractions: F1 = 11.0–7.0 µm, F2 = 7.0–4.7 µm, F3 = 4.7–3.3 µm, F4 = 3.3–2.1 µm, F5 = 2.2–1.1 µm, F6 = 1.1–0.65 µm

In spring and autumn, there were more molds than yeasts in the air of the studied stables (Table 7) and at the control station (Table 8). The share of mold in this period ranged from 69.8 to 86.1%. In turn, in summer and winter, the air in the studied stables was dominated by yeast, whose share in these periods ranged from 60.3 to 71.4%.

**Table 7:** Share molds and yeasts depending on the season [%]

Stable	Spring (April)		Summer (July)		Autumn (September)		Winter (February)	
	Molds	Yeasts	Molds	Yeasts	Molds	Yeasts	Molds	Yeasts
All R	69.8	30.2	38.4	61.6	79.8	20.2	28.6	71.4
All B	78.6	21.4	39.7	60.3	86.1	13.9	33.3	66.7
C	84.8	15.2	73.5	26.5	80.3	19.7	37.7	62.3

**Table 8:** Inside/outside ratio (I/O) of bioaerosol concentrations depending on the season and the horse care system

Stable	Spring (April)		Summer (July)		Autumn (September)		Winter (February)	
	BB	AB	BB	AB	BB	AB	BB	AB
MOLDS								
All R	20.3	9.3	10.2	5.9	8.9	8.7	38.2	6.6
All B	29.8	12.9	4.0	1.9	15.5	11.3	15.9	6.7
YEASTS								
All R	53.7	20.1	88.9	13.0	14.1	10.1	117.4	12.5
All B	37.9	10.8	30.7	10.3	18.4	6.4	28.6	6.2

An important factor indicating the occurrence of mycotoxin contamination is the index showing the degree of intoxication (inside/outside (I/O)) of the air in stables (Table 8); this phenomenon occurs when the concentration of microorganisms in the air inside the stable is higher than that in the atmospheric air outside the stable. High levels of intoxication caused by molds and yeasts were recorded especially in the winter before the stables cleaning (in the case of molds, they were up to 90 times higher compared to the control, and in the case of yeasts, 135 times higher). In winter, higher levels of intoxication were found in the runners. Slightly lower levels of intoxication (up to approximately 37 ×) in the case of molds and 59 × in the case of yeasts) were recorded in spring. The lowest level of intoxication occurred in the case of mold in the runners in autumn (on average about 9 ×).

When testing a particulate matter level in the stables, it was found that the highest concentrations were those of fraction PM_10_: in both types of stables, as well as at the control station (Table 9). A significant increase in the particulate matter level after cleaning the runners, ranging from 56.2% (fraction PM_1_) to 110.8% (PM_10_), is noteworthy. Dust particles in the stable air behave differently from fungal spores and yeast, the concentrations of which were lower after cleaning the stables; this proves their separate occurrence in the air. In the box stables, the increase of particulate air pollution was much lower than in the runners and ranged from 1.5 to 14.5% depending on a fraction. In the runners, after cleaning, the concentration of fraction PM_1_ increased the least, and PM_10_ the most; in the box stables, the trend was the opposite.

**Table 9:** Particulate matter divided into fractions [µg/m^3^]

All R					
	BB bedding		AB bedding		
Particulate matter	Median ± standard deviation	Range	Median ± standard deviation	Range	Change in relation BB vs BB to the median particulate matter concentrations [%]
PM10	157 ± 115	39–636	331 ± 234	64–1,110	110.8
PM4	150 ± 73	36–298	262 ± 143	54–592	74.7
PM2.5	132 ± 73	33–314	229 ± 116	49–454	73.5
PM1	130 ± 66	28–258	203 ± 162	39–847	56.2
All B					
PM10	203 ± 85	49–370	206 ± 153	140–740	1.5
PM4	189 ± 76	43–318	192 ± 88	120–412	1.6
PM2.5	176 ± 71	38–282	183 ± 78	110–387	4.0
PM1	152 ± 65	32–244	174 ± 68	66–321	14.5
Control (C)					
	Median ± standard deviation	Range			
PM10	113 ± 53	41–191			
PM4	108 ± 56	43–189			
PM2.5	106 ± 61	42–196			
PM1	96 ± 61	31–198			

The impact of PM on the concentration of molds and yeast aerosols was assessed using Spearman’s rank correlation coefficient (Table 10). The analysis showed no significant correlation between the concentration of mold and the particulate matter of fractions: PM_1.0_, PM_2.5_, PM_4.0_, and PM_10.0_ (correlation coefficients respectively: *R* = 0.19, *R* = 0.20, *R* = 0.19, *R* = 0.18). The analysis also showed no significant correlation between the concentration of yeast and the particulate matter of fractions: PM_1.0_, PM_2.5_, PM_4.0_, and PM_10.0_ (correlation coefficients respectively: *R* = 0.14, *R* = 0.11, *R* = 0.14, *R* = 0.16).

**Table 10:** Spearman’s rank correlation analysis between particulate matter and fungal aerosol concentration

Factor	R-Coefficient for mold aerosol concentration	R-Coefficient for yeast aerosol concentration
PM1	0.19 ns	0.14 ns
PM2.5	0.20 ns	0.11 ns
PM4	0.19 ns	0.14 ns
PM10	0.18 ns	0.16 ns

^*^ns: correlation coefficient not significant for *p* < 0.05

## Discussion

Air is a specific environment characterized by high variability of parameters, including those subject to mycological research. Measurement of the concentration of microorganisms taken in a specific environment characterizes its contamination at a specific moment (a specific point in time). Difficulties in interpretation are also caused by the lack of appropriate regulations regarding microbiological air quality. The absence of regulations is the result of, among other things, the lack of a simple relationship between the absorbed dose of bioaerosol and the health effect, but also the lack of a sufficient number of studies performed in various environments. The obtained research results are therefore related to the proposed norms drawn up by ZECB (Augustyńska and Pośniak 2016). There are not many studies on the mycological quality of air in stables, and an additional problem in comparing the obtained results is the use of different measuring devices.

Mold fungi and their morphotic elements can sensitize, can infect, and may have a toxic effect on both stable workers and horses (Górny and Dutkiewicz 2002). Dauvillier et al. (2018) proved that horses inhale fungal spores and mycelium fragments that are part of bioaerosol and that mold fungal elements have been found in horses respiratory tracts; this causes a significant increase in exposure to inflammatory airway disease (IAD) and RAO (recurrent equine obstructive disease). Fungi are not necessarily the primary cause of IAD, but when immune function is compromised (e.g., due to intense training or transport stress), they may affect horses’ ability to respond to infection. Similarly, in the case of healthy workers handling horses in stables, the morphotic elements of fungi may have a minor impact on their health, but factors such as a weakened immune system and treatment with corticosteroids or antibiotics may change it (Perrin 2021).

Mold concentrations in stables can vary significantly. This is influenced by many factors, such as location, type of stable, livestock density, type of ventilation, type of bedding, and frequency of stable cleaning (Budzińska et al. 2016). In our studies, mold concentrations in the box stables ranged from 1700 to 81,000 CFU/m^3^, while in the runners they ranged from 797 to 255,777 CFU/m^3^. The most similar values of fungal aerosol concentrations in box stables were found by Lenart-Boroń et al. (2022) (1370–112,100 CFU/m^3^) and Wålinder et al. (2011) (box stables: 200–96,000 CFU/m^3^). Budzińska et al. (2016) recorded several times lower fungi concentrations, ranging from 1270 CFU/m^3^ in winter to 47,800 CFU/m^3^ in autumn. A similar concentration range was shown by Dutkiewicz et al. (1994) (1700–28,000 CFU/m^3^). Even lower concentrations of airborne molds were observed by Witkowska et al. (2012) (1000–10,000 CFU/m^3^), and Wolny-Koładka (2018) (100 to 2400 CFU/m^3^).

Depending on the location of a given stable, different microclimatic conditions prevail inside each of them, which affects the fungi concentration. The distribution pattern of mold concentrations during different seasons may also vary (Auger and Moore-Colyer 2017; Nazarenko et al. 2018). Comparing our results with those obtained by Wolny-Koładka (2018), it was found that the highest mold concentrations in both cases were in summer (July), but the lowest were different: autumn (September) vs. winter (February). Both studies were performed in southern Poland.

When analyzing the distribution of mold spores in stable air, it was found that their largest share was recorded for the fine fraction F5 (2.2–1.1 µm), but the highest concentrations were in the range 4.7–1.1 µm (fractions F3–F5). The significant predominance of mold spores belonging to fraction F5 was confirmed by studies by Lenart-Boroń et al. (2022), as well as in these studies, fine and very fine fractions dominated (3.3–0.65 µm). Different results were presented by Mostafa et al. (2021), in whose study the highest concentrations of fungal spores were recorded for fractions ranging from > 11 to 2.1 µm, although they differed depending on the type of mold (*Cladosporium* > 11–2.1 µm, *Wallemia* 4, 7–2.1 µm, *Eurotium* > 11–4.7 µm, *Aspergillus* > 11–2.1 µm). It should be noted that these research were performed throughout the year (12 months) in the North Rhine-Westphalia (Germany).

Morphotic elements of fungi (mycelium fragments and spores) can float in the air on their own, but can also be attached to dust particles; therefore, when performing tests in a specific facility, it is important to simultaneously assess a particulate matter concentration. In our studies, we recorded PM_10_ concentrations in the box stables ranging from 49 to 740 µg/m^3^, while in the runners the range of particulate matter from this fraction was larger and ranged from 39 to 1110 µg/m^3^. Similar PM_10_ particulate matter concentrations were recorded in box stables by Wolny-Koładka (2018) 50–748 µg/m^3^. A much smaller range of concentrations, and at the same time several times lower maximum concentrations, were noted by Nazarenko et al. (2018) (62.8–125.7 µg/m^3^). In the case of fraction PM_2.5_, the concentration range in our studies was narrower than in Wolny-Koładka (2018) (38–387 µg/m^3^ vs. 48–654 µg/m^3^). The highest levels of particulate matter in both studies were found during the winter season. In autumn and winter, the type of fuel used to heat residential premises located in the vicinity of the stables, which are a subject of our studies, has a significant impact on the maximum concentrations of PM in the air. As in the case of fraction PM_10_, also for the finer fraction, PM_2.5_, Nazarenko et al. (2018) recorded the lowest particulate matter concentrations when straw was used as bedding (7–10 µg/m^3^), although it is believed that straw significantly affects the level of particulate matter, as proved by our research. Clements and Pirie (2007) found three times higher PM_4_ concentrations when straw was used as bedding compared to when bedding consisted of wood shavings.

In comparison to the results obtained by Lenart-Boroń et al. (2022), our results regarding particulate matter concentration in the box stables were at least two times higher; we recorded PM_10_ concentrations from 203 to 206 µg/m^3^ and for PM_2.5_ 189–192 µg/m^3^. Values obtained by Lenart-Boroń et al. (2022) for PM_10_ were 10–109 µg/m^3^, and for fraction PM_2.5_, 9–86 µg/m^3^.

In our research, yeasts dominated the air in both types of stables (box stables and runners) in two seasons (summer and winter). The share of yeast in the total number of molds and yeasts ranged from 13.9 to 71.4%. No studies have been found in the available literature to compare yeasts and molds in stables. In studies carried out in hen houses, Sowiak et al. (2012) showed that yeast accounted for only 12% of all fungi, which was below the lower limit of the yeast share in our studies.

Polish legislation provides for the permissible concentration of PM_10_ and PM_2.5_ in the atmospheric air, but the limit value for the concentration during 1 day is only for PM_10_; it is 50 µg/m^3^. For fraction PM_2.5_, the pollution threshold refers to the calendar year. While taking most of the measurements, especially after cleaning the stables, the permissible concentration of PM_10_ was exceeded. According to Fiedorowicz (2007), the maximum particulate matter level in the stable cannot exceed 3 mg/m^3^; considering this value, the permissible PM_10_ concentration in the tested stables was not exceeded.

The conducted statistical analyses did not show a significant correlation between the concentrations of the tested microorganisms with the concentrations of any of the tested particulate matter fractions.

The conducted studies allowed for the conclusion that there is a high level of air intoxication in stables, both by yeast and by mold fungi. It has been proven that horses kept in box stables are exposed to lower concentrations of molds and yeasts (lower intoxication) compared to runners (Wolny-Koładka 2018). In spring and autumn, i.e., in the wetter periods, it was recorded that molds predominated in the stables, while in summer and winter yeasts prevailed. The fewest exceedances of the permissible concentration of fungi (in total: molds and yeasts) in the stable air occurred in autumn (TC and RF), while the highest levels for TC occurred in summer, and for RF in spring. It was observed that cleaning stables reduces the number of fungal morphotic elements in the air, even though during this process the level of particulate matter in stables increases. In the case of runners, the increase in PM is much higher than that in box stables. It should be emphasized that at such concentrations of fungi as determined during our studies carried out in the stables, fungal morphotic elements of fungi may pose a potential health threat to horses, staff, and people using horses.

## Data Availability

The datasets used and/or analyzed during the current study are available from the corresponding author on reasonable request.
